# A Pliocene Precipitation Isotope Proxy‐Model Comparison Assessing the Hydrological Fingerprints of Sea Surface Temperature Gradients

**DOI:** 10.1029/2021PA004401

**Published:** 2022-12-24

**Authors:** Scott Knapp, Natalie J. Burls, Sylvia Dee, Ran Feng, Sarah J. Feakins, Tripti Bhattacharya

**Affiliations:** ^1^ Department of Atmospheric, Oceanic, and Earth Sciences George Mason University Fairfax VA USA; ^2^ Department of Earth, Environmental and Planetary Sciences Rice University Houston TX USA; ^3^ Department of Geosciences University of Connecticut Storrs CN USA; ^4^ Department of Earth Sciences University of Southern California Los Angeles CA USA; ^5^ Department of Earth and Environmental Sciences Syracuse University Syracuse NY USA

**Keywords:** Pliocene, hydrological cycle, plant wax, hydrogen isotopes

## Abstract

The Pliocene offers insights into future climate, with near‐modern atmospheric pCO_2_ and global mean surface temperature estimated to be 3–4°C above pre‐industrial. However, the hydrological response differs between future global warming and early Pliocene climate model simulations. This discrepancy results from the use of reduced meridional and zonal sea surface temperature (SST) gradients, based on foraminiferal Mg/Ca and Alkenone proxy evidence, to force the early Pliocene simulation. Subsequent, SST reconstructions based on the organic proxy TEX_86_, have found warmer temperatures in the warm pool, bringing the magnitude of the gradient reductions into dispute. We design an independent test of Pliocene SST scenarios and their hydrological cycle “fingerprints.” We use an isotope‐enabled General Circulation Model, iCAM5, to model the distribution of water isotopes in precipitation in response to four climatological SST and sea‐ice fields representing modern, abrupt 4 × CO_2_, late Pliocene and early Pliocene climates. We conduct a proxy‐model comparison with all the available precipitation isotope proxy data, and we identify target regions that carry precipitation isotopic fingerprints of SST gradients as priorities for additional proxy reconstructions. We identify two regions with distinct precipitation isotope (D/H) fingerprints resulting from reduced SST gradients: the Maritime Continent (D‐enriched due to reduced convective rainfall) and the Sahel (wetter, more deep convection, D‐depleted). The proxy‐model comparison using available plant wax reconstructions, mostly from Africa, is promising but inconclusive. Additional proxy reconstructions are needed in both target regions and in much of the world for significant tests of SST scenarios and dynamical linkages to the hydrological cycle.

## Key Points

- Climate model experiments forced with Pliocene‐like sea surface temperatures yield distinct regional precipitation isotope fingerprints
- All available Pliocene plant wax hydrogen isotope proxy evidence of precipitation isotopes are compared to isotope‐enabled simulations
- Most proxy data from around Africa, yet more data needed in the Sahel as well as global data voids including the maritime continent

## 1 Introduction

Global warming is predicted to exaggerate the modern patterns of precipitation minus evaporation, such that the tropics get wetter and the subtropics get drier under what is referred to as the “thermodynamic effect” (Held & Soden, 2006; Seager et al., 2010). Assuming relative humidity remains roughly the same, an assumption that does not hold well over land (Byrne & O’Gorman, 2015), the Clausius–Clapeyron relation predicts 7% more water vapor per degree Celsius of warming. If atmospheric circulation remains unchanged, this thermodynamic effect results in the more efficient transport of moisture from the subtropics into the tropics. However, a slight reduction of large‐scale circulation strength, the “dynamic effect,” is predicted to partially counteract this thermodynamic effect (Held & Soden, 2006; Seager et al., 2010). Changes to the hydrological cycle predicted by climate models appear dominated by the thermodynamic effect in response to near future warming (Seager et al., 2010) and an abrupt quadrupling of pre‐industrial CO_2_ levels (Burls & Fedorov, 2017). Results from Phase 6 of the Coupled Model Intercomparison Project (CMIP6) predict global monsoon precipitation will increase by the end of the twenty‐first century (Wang et al., 2021). CMIP6 results also predict that regional changes in precipitation over the oceans will be affected by the uneven heating of the ocean surfaces (Xie, 2020).

A complementary perspective on how the hydrological cycle might change under global warming can be gained by examining past warm climates, such as the Pliocene. The Pliocene (5.3–2.6 million years ago; Mya) had a similar continental configuration and included times when atmospheric pCO_2_ approached modern values (∼400 ppm) (Martínez‐Botí et al., 2015). Global mean surface temperature (GMST) estimates from reconstructions of deep ocean temperature indicate an early (∼4–5 Mya) Pliocene GMST about 3°C warmer than pre‐industrial, cooling by about 1–2°C in the late (∼3 Mya) Pliocene (Hansen et al., 2013). These GMST estimates make both the late (Burke et al., 2018) and early (Burls & Fedorov, 2017) Pliocene potential analogs for future global warming scenarios.

The Pliocene provides an interesting test of our understanding and simulations of how the hydrological cycle may change in a warmer climate. A long‐term cooling trend in SSTs in the mid‐to high‐latitudes and upwelling zones over the last 5 million years emerges as a robust signal in reconstructions (Fedorov et al., 2013; Herbert et al., 2016). However, there is still considerable debate as to just how much warmer the SSTs were in the Indo‐Pacific Warm Pool (IPWP) (Fedorov et al., 2013; O’Brien et al., 2014; Ravelo et al., 2006; Tierney et al., 2019; Zhang et al., 2014). With Mg/Ca records suggesting lower temperatures and a reduced zonal gradient, whereas TEX_86_ suggests warmer temperatures and a zonal gradient more similar to modern. Model experiments have tested the effects of reduced meridional and zonal SST gradient scenarios for the Pliocene (e.g., Brierley & Fedorov, 2010). In addition, a recent modeling study, Burls and Fedorov (2017), shows that the reduced zonal and meridional SST gradients of the early Pliocene could have supported wetter subtropics and drier tropics via a weakening of the Hadley circulation, allowing the dynamic effect to overcome the thermodynamic effect. These conditions are supported by paleobotanical data, which show an expanse of savannas and woodlands in Africa and Australia and a reduction of deserts during the mid‐to late Pliocene (Salzmann et al., 2008), although recent studies demonstrated that terrestrial warming patterns due to vegetation and icesheet changes may have also played an important role in driving summer precipitation across North Africa and east Asia (Feng et al., 2022). During the warmer early Pliocene, relatively wet conditions are apparent in eastern Africa before cooling and drying (Liddy et al., 2016). There is also evidence for wetter subtropical regions in the warm late Miocene (Pound et al., 2011) when SST gradients were similarly depressed (Herbert et al., 2016). Paleobotanical evidence of subtropical wettening is however largely qualitative with regards to the hydrological cycle.

Precipitation isotopic proxies, namely plant wax δD, are typically recovered in marine sedimentary archives for the Pliocene (e.g., Huang et al., 2007) and more rarely in lake sedimentary archives (Lupien et al., 2020). They offer quantitative tracing of the hydrological cycle, such that precipitation isotope data can be directly compared to isotope‐enabled climate model experiments. Here, we simulate stable hydrogen and oxygen isotopes in precipitation (δD and δ^18^O) in response to early Pliocene‐like SST forcing and compare with other warming scenarios which are more strongly controlled by thermodynamic effects. This initial precipitation δD proxy‐model comparison aims to identify regions where dynamic effects are consistently represented in water isotopes, in order to motivate new proxy reconstructions to better inform future model‐proxy comparisons.

Water isotopes are valuable tracers in the hydrological cycle, as their concentration is the result of many processes. The preservation of water isotopes over geological time periods presents the opportunity to reconstruct snapshots of the hydrological cycle in previous climates. We focus on the tropics, where changes to the Hadley and Walker circulations caused by different SST gradients should be reflected in changing water isotope ratios. An observed negative correlation between water isotope ratios and rainfall amount in the tropics is known as the “amount effect” (Dansgaard, 1964). The amount effect is not well understood, but a number of processes are thought to contribute to the overall alteration of water isotope ratios during rainfall events (Galewsky et al., 2016; Lee & Fung, 2008). The amount of reevaporation of falling raindrops—a process which enriches the isotopic ratio in the raindrops—can be reduced by increasing droplet radius or increasing relative humidity (Dansgaard, 1964; Lee & Fung, 2008; Risi et al., 2008), both of which are common in deep convective precipitation. The isotope ratios in the surrounding vapor are reduced by the condensation of precipitation, as well as the vertical mixing and recycling of vapor in convective systems (Risi et al., 2008 and see Galewsky et al., 2016 for a review of water isotopes in the atmosphere).

Water isotopes have been successfully integrated into the hydrological cycle of several modern general circulation models (GCM) (Lee et al., 2007; Schmidt et al., 2007). We employ the isotope enabled Community Atmosphere Model (iCAM5.3; Brady et al., 2019) with climatological SST and sea‐ice fields representative of the early (Burls & Fedorov, 2014b) and late Pliocene (Feng et al., 2020), along with an abrupt 4 × CO_2_ future warming scenario run to near equilibrium (3,000 years) (Burls & Fedorov, 2017). The 4 × CO_2_ scenario is included to represent how future global warming might change SSTs, given a global mean surface temperature increase comparable to the early Pliocene. In this 4 × CO_2_ scenario, thermodynamic effects on water vapor content dominates over any atmospheric dynamic changes. The early Pliocene (EP model scenario) climatology is meant to represent the average conditions from a broad period of time 4–5 Mya. The late Pliocene (LP) climatology is the response of a coupled model to the PRISM4 boundary conditions (Dowsett et al., 2016; Feng et al., 2020), following the PlioMIP2 protocol (Haywood et al., 2016), meant to represent conditions during a narrow time slice centered on 3.2 Mya. The key distinction between the three warming scenarios are their SST gradients. The 4 × CO_2_ and LP SST fields have zonal and meridional gradients which are similar, despite the GMST being much warmer in the 4 × CO_2_ experiment. The EP scenario has far weaker zonal and meridional SST gradients than both the 4 × CO_2_ and late Pliocene. By comparing the results of the three experiments, we aim to identify regional fingerprints where dynamic effects are consistently represented in simulated water isotope fields, to inform interpretation of future model‐proxy comparisons.

We identify two sub‐continental scale regions of land (the maritime continent and Sahel) where δD of precipitation (δDp) is distinct between the 4 × CO_2_ and late Pliocene scenarios. We investigate how these two regional features result from dynamics driven by prescribed SST gradients. We compare proxy and model results globally, but almost all available data is for Africa, with additional data from the Bengal Fan, and no Pliocene δD data in the rest of the world. Proxy‐model comparisons are promising, appearing to support the model results, although selection of the best fit model scenario is inconclusive at present. We show more δDp proxy data are needed in Africa (primarily more temporal density to assess variability, and add confidence in reconstructions) and spatial coverage across the rest of the world (where data is currently lacking). More proxy data would enable more conclusive comparisons of model scenarios including the questions over the zonal Pacific SST gradient strength emphasized here. We recommend prioritizing data collection where models indicate sensitivity to large‐scale SST patterns; however, other regional hydroclimate motivations (e.g., marginal or populous regions) should also be considered to improve our understanding of hydroclimate in a warm climate state of the recent past.

## 2 Methods

### 2.1 Isotope Enabled Atmospheric GCM

This study employs the isotope‐enabled Community Atmosphere Model (iCAM) version 5.3 (Nusbaumer et al., 2017). The model was run with prescribed SST and sea‐ice, so that the only active model components are the atmosphere and the land. The land model, iCLM4, is the isotope enabled version of the Community Land Model version 4 (iCLM4), described in Wong et al. (2017). In both models, water isotopologue masses are explicitly simulated and tracked through the hydrological cycle as conservative tracers.

Nusbaumer et al. (2017) ran iCAM5 coupled with iCLM4 over the years 1975–2014 to compare its fidelity simulating isotope ratios to observations from the Global Network for Isotopes in Precipitation (GNIP) and reanalysis from ERA‐Interim. Compared to reanalysis, the model is biased toward higher global mean specific humidity (+0.23 g/kg) and lower GMST (−1.59 K), along with a slight global mean precipitation bias (+0.13 mm/day) which was largest over oceans and negative over some tropical land regions. There is also a large positive global mean evaporative flux bias (+0.16 mm/day), especially over subtropical oceans. The model values for the isotopic content of precipitation are generally too negative compared to GNIP observations, with a global mean model δD bias of −20‰. However, the majority of GNIP stations are in Europe, and the observational data provide an imperfect test of model fidelity for many regions of the globe. The same model iCAM5 was run using 1.9**°** × 2.5**°** horizontal resolution for the historical observation comparisons (Nusbaumer et al., 2017), while higher resolution (0.9**°** × 1.25°) was used for our Pliocene study. The higher resolution should improve fidelity, although a historical comparison has not been performed. Despite these documented biases, iCAM5 generally captures the large‐scale spatial and temporal trends of global water isotope ratios.

### 2.2 Model Configuration

The model is run with prescribed climatological SST and sea‐ice and a pre‐industrial component set (F_1850_CAM5), that is, atmospheric greenhouse gas and aerosol concentration set to preindustrial values. Modern geography and vegetation are used for all experiments, given our focus on testing the sensitivity of the hydrological cycle to different SST gradients. Ocean isotope ratios are prescribed based on modern values (Nusbaumer et al., 2017). pCO_2_ is prescribed as 284 ppmv for all runs, but the prescribed SST warming in the below‐described experiments leads to elevated water vapor concentrations and an associated greenhouse effect. The atmosphere is run at 0.9**°** × 1.25**°** horizontal resolution with 30 vertical layers. The ocean and sea‐ice are on the gx1v6 grid. Each simulation is run for 100 years, with the last 50 years averaged for analysis.

### 2.3 Experiments

Four simulations are run using iCAM5.3, forced by monthly climatological SST and sea ice fields. All simulations use preindustrial land surface conditions. The four experiments are: pre‐industrial control (PIC), abrupt 4 × CO_2_ (A4X), early Pliocene (EP), and late Pliocene (LP). The PIC SST and sea ice are the average of years 1870–1890 from the observational data set described in Hurrell et al. (2008). The A4X conditions represent a pre‐industrial climate suddenly forced with a four‐fold increase in atmospheric CO_2_ and run for 3,000 years to near‐equilibrium. The coupled climate model simulation from which the A4X SST and sea ice climatology is derived is described in Burls and Fedorov (2017). The EP SST and sea ice fields are taken from a coupled climate model simulation chosen from a suite of perturbed cloud‐albedo experiments, namely Experiment 16 from Burls and Fedorov (2014a). This climatology was selected because it best represents features seen in ocean‐based SST proxies from 4 to 5 million years ago, such as greatly reduced zonal and meridional SST gradients (Burls & Fedorov, 2014b). The LP SST and sea ice climatologies represent the simulated response of a coupled model to reconstructed boundary conditions for the mid‐Piacenzian warm period 3.2 million years ago. The mid‐Piacenzian was formerly referred to as the mid‐Pliocene before the definition of the Pliocene was changed in 2009 (Gibbard & Head, 2010). The LP SST and sea ice climatologies were obtained by running a fully coupled CESM1.2 simulation with the PRISM4 boundary conditions (Dowsett et al., 2016; Feng et al., 2020), following the PlioMIP2 protocol (Haywood et al., 2016). In addition to CESM1.2, Feng et al. (2020) also tested the Community Climate System Model version 4 and CESM2 with the PlioMIP2 protocol and found that CESM1.2 agrees best with proxy SST data.

**Figure 1:**
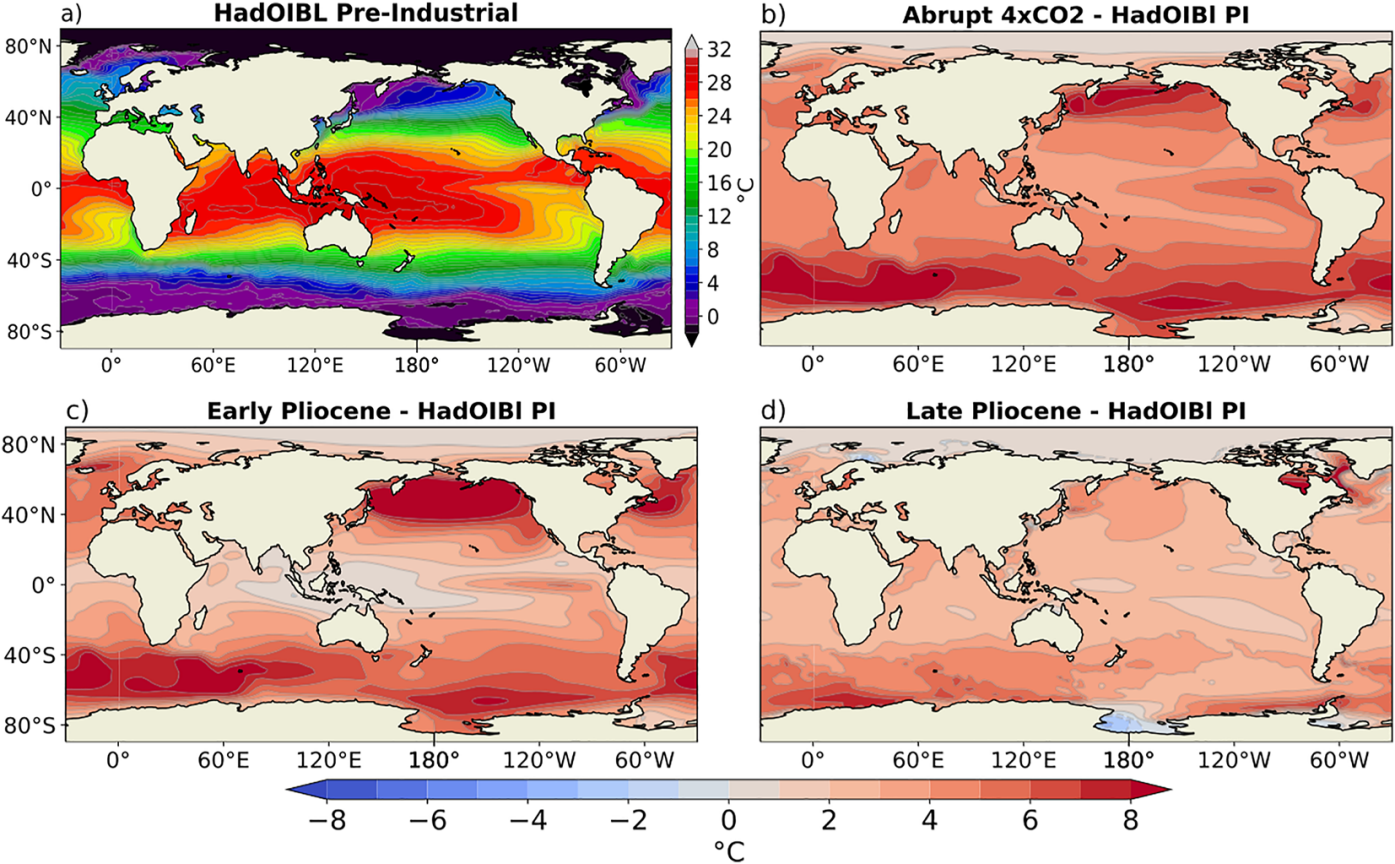
(a) Pre‐industrial control (PIC) annual mean SST field. (b)–(d) Warming scenario annual mean SST minus PIC for (b) the A4X, (c) EP and (d) LP experiments.

In order to create a common control, the three warm scenarios, A4X, LP, and EP, were translated into anomaly fields by subtracting their respective coupled climate model PI control monthly climatological SST and sea‐ice fields. These anomalies were then added to the PIC monthly climatological SST and sea‐ice fields. The resulting 12‐month SST and sea‐ice fields were used as boundary conditions in this study. The annual mean SST derived from the monthly PIC climatology is shown in Figure 1a, and the three annual mean SST anomaly fields are shown in Figures 1b–1d.

### 2.4 Idealized Rayleigh Column

In order to differentiate between changes in δD of water vapor (δD_v_) due to thermodynamic changes and those due to changes in atmospheric dynamics, an idealized single‐column Rayleigh distillation model is employed. This model, described in Dee et al. (2018), simulates the effects of Rayleigh distillation on a saturated parcel of air which rises and cools. For each grid cell, the δD_v_ is initialized at −80‰, to represent near‐surface air that is near equilibrium with the ocean. The air parcel is assumed to be saturated at each vertical level and cools according to the vertical temperature and pressure profiles from iCAM5. As it cools, condensation is removed via Rayleigh distillation, depleting the vapor. The predicted δD_v_ due to Rayleigh distillation alone is called the thermodynamic term and represents the maximum theoretical depletion due to the vertical temperature profile. Although dynamic changes in each model are reflected in the temperature profile, our thermodynamic term is meant to isolate the effect of temperature on isotopes. The residual between the thermodynamic term and the δD_v_ from iCAM5 is called the dynamic term and represents the effects of atmospheric advection on δD_v_.

As the key assumption made with this single‐column model is that the atmospheric lapse rate is a pseudoadiabat, it is most accurate in the humid tropics (Dee et al., 2018). For drier unsaturated areas, the assumed thermodynamic effect will overestimate the depletion from condensation. For this reason, we only employ this model for analysis in the tropical Pacific.

### 2.5 Proxy Data Synthesis

We conduct a model‐proxy comparison between the available Pliocene‐aged estimates of precipitation isotopic composition (*n* = 8 marine cores and *n* = 1 lake core) and each of the four iCAM simulations. We surveyed the literature for plant wax hydrogen isotope reconstructions from marine and lake cores spanning two periods of interest: 5–4 Ma (early Pliocene), and 3.3–3.1 Ma (late Pliocene). Within the available Pliocene δD timeseries, the number of samples within the time slices of interest for the model comparisons are as high as *n* = 41 (Liddy et al., 2016) but are typically much lower, and in many cases the data density precludes any constraint or assessment of whether temporal variability such as associated with glacial‐interglacial or precessionally paced precipitation changes might affect the mean result. Other questions such as age model sufficiency were not considered or updated for this initial comparison of the available data, as the low data density clearly precluded opportunities for selectivity or improvement, and we wanted to include all available data. We considered various schemes for interpreting precipitation isotopes from the proxy, and settled upon a standardized approach given the variable availability of additional constraining data such as plant wax 𝛿^13^C and pollen data, as well as climatic differences between tropical rainforest and desert environments (see discussion in the Supporting Information S1). Plant wax 𝛿D were corrected for a constant apparent fractionation of −100‰ to yield an inferred 𝛿D of precipitation for each site and these were compared to the model estimate from the inferred plant wax source regions based on consideration of geological context and dominant transport mechanism (see discussion in the Supporting Information S1).

## 3 Results

**Figure 2:**
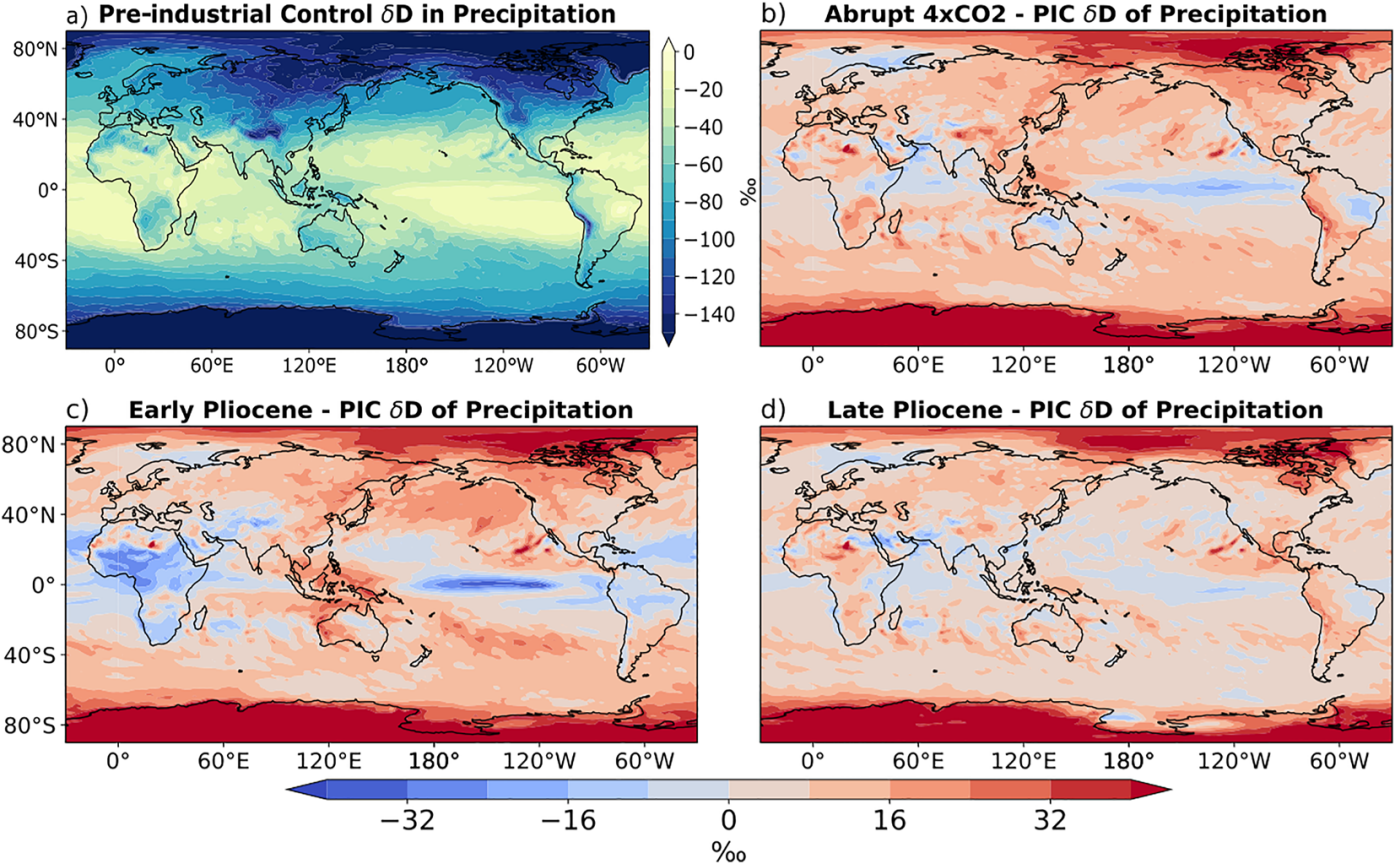
(a) Annual mean 𝛿D of precipitation in pre‐industrial control (PIC). (b)–(d) Warming scenario annual mean 𝛿D of precipitation minus PIC for (b) the abrupt 4 × CO_2_, (c) early Pliocene, and (d) late Pliocene experiments.

**Figure 3:**
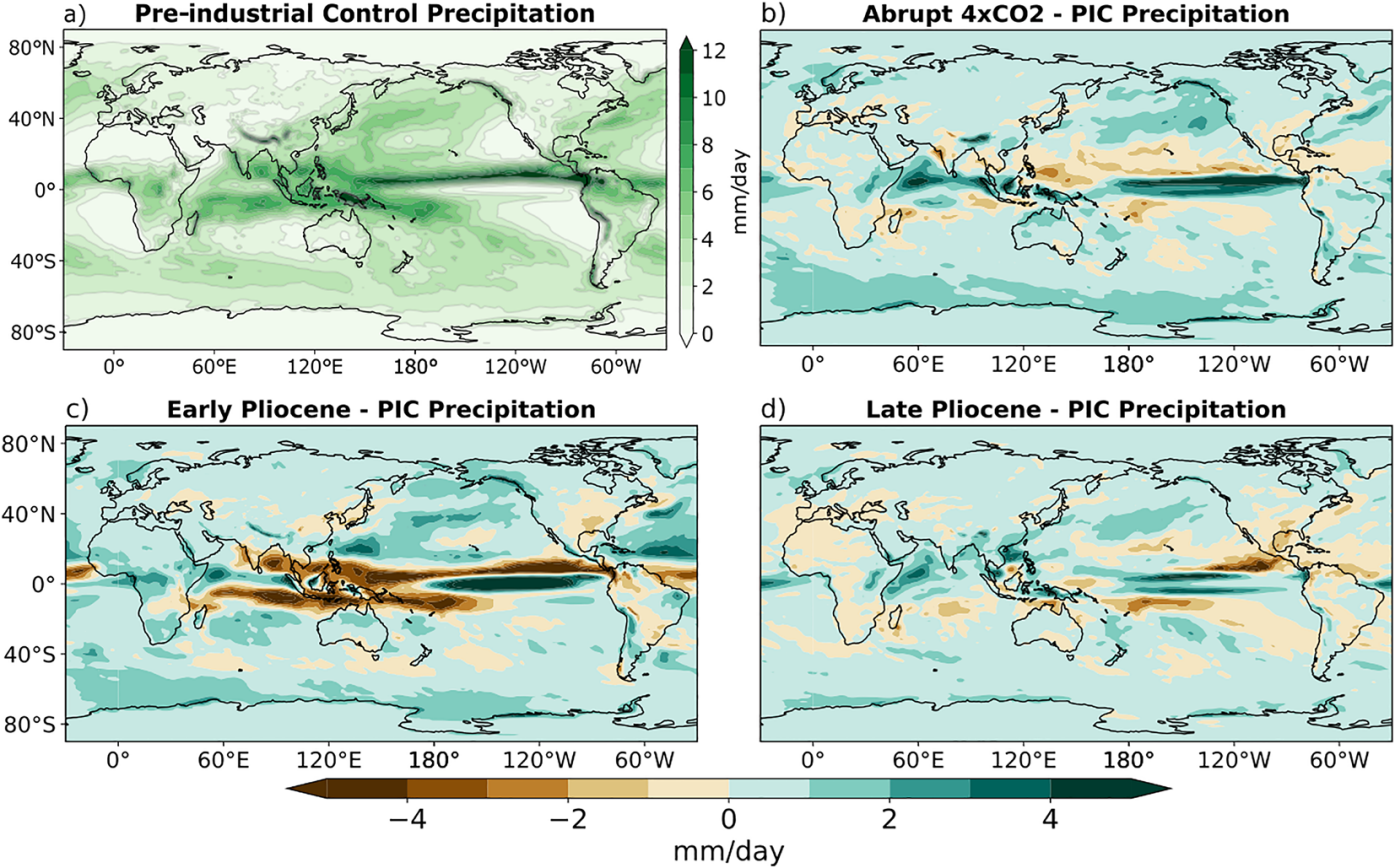
(a) Annual mean daily precipitation in pre‐industrial control (PIC). (b)–(d) Annual mean daily precipitation in warming experiments minus PIC: (b) Abrupt 4 × CO_2_—PIC, (c) early Pliocene—PIC, (d) late Pliocene—PIC.

The PIC experiment captures the key features seen within the modern δD_p_ field (Figure 2a). The Intertropical Convergence Zone (ITCZ) is characterized by a band of consistent isotopic depletion, with more extreme depletion over the IPWP (Figure 2a). These are clear examples of the amount effect, where heavy convective precipitation drives more negative isotopic values (Figure 3a). The temperature effect dominates at higher latitudes, exhibiting a general trend toward more negative δD_p_ moving poleward. Continental depletion due to Rayleigh distillation is most obvious moving inland over North America and Asia.

Relative to the PIC, there is an increase in δD_p_ in most regions in all three warming experiments (Figures 2b–2d). This is expected due to the global warming signal, but is most prominent in polar regions due to the clear polar amplification seen in all three experiments (Figure 1). Common areas of lower δD_p_ are present in all three warming scenarios as well, such as in the central Pacific and western Indian oceans, which coincide with areas of increased precipitation relative to the PIC (Figure 3).

**Figure 4:**
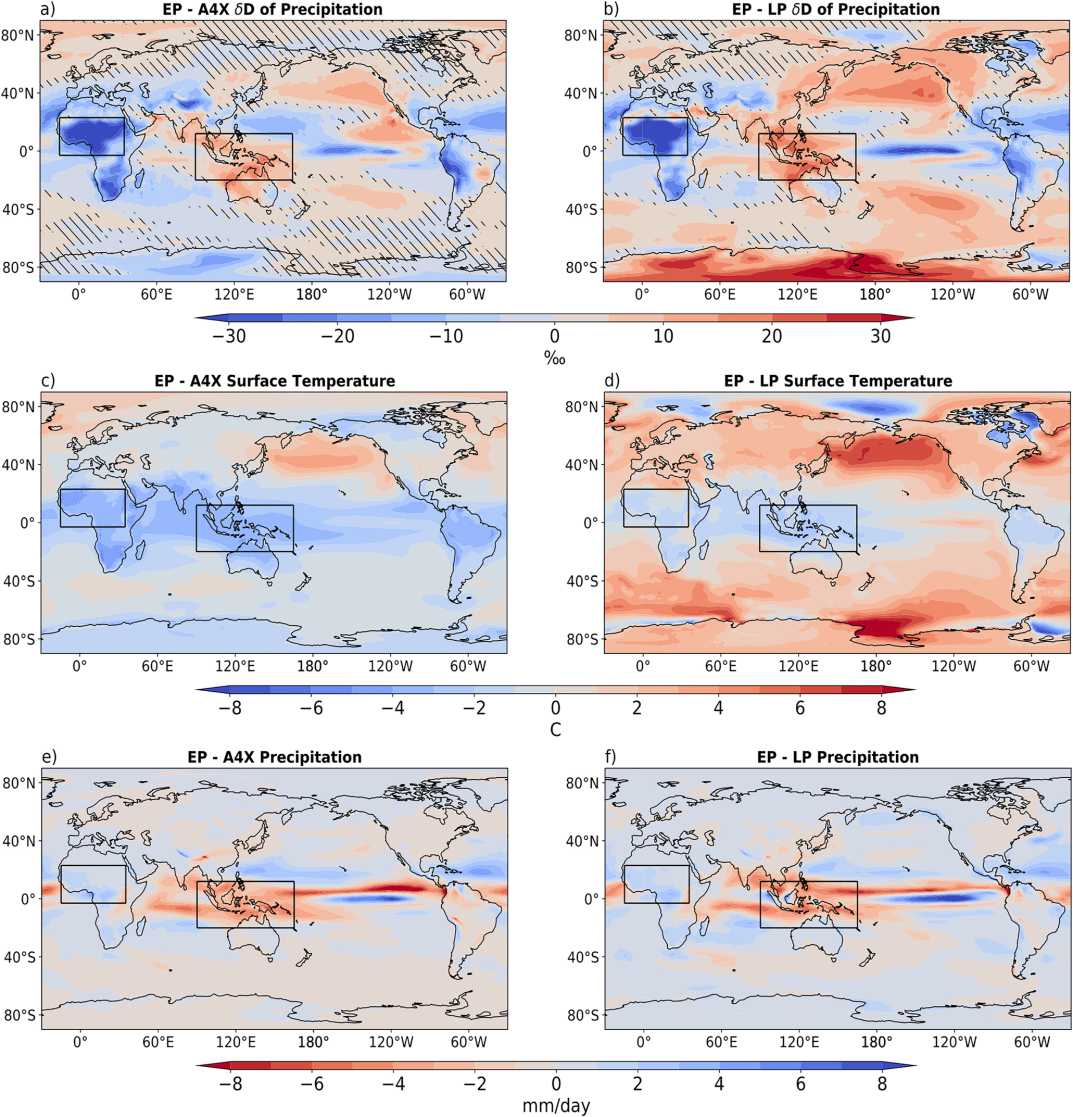
(a) and (b) show differences in 𝛿D of precipitation between the early Pliocene and 4 × CO_2_ and the early and late Pliocene, respectively. Hatched areas indicate values less than two standard deviations from a set of 10 20‐year means taken from a 200‐year control run. (c) and (d) show differences in surface temperature, and (e) and (f) show differences in precipitation. All values are annual means. Black boxes outline the two regions of Early Pliocene fingerprints.

In order to visualize the regions in which the weakened SST gradients of the EP result in a δD_p_ signal which is most distinct from the SST gradients of the LP and A4X, the differences are plotted in Figures 4a and 4b. Figures 4c and 4d illustrate the difference in the warming patterns between the EP experiment and the A4X and LP experiments respectively. In both cases it is evident that the meridional and zonal SST gradients are much weaker in the EP experiment (also see Table 1). Comparing Figure 4a with Figure 4c and Figure 4b with Figure 4d, many of the high latitude δD_p_ features coincide with differences in the degree of regional warming; higher temperatures correspond to higher δD_p_, and lower temperatures correspond to lower δD_p_ (temperature effect; Dansgaard, 1964). In the tropics, decreased precipitation in the EP relative to both A4X and the LP generally coincides with an increase in δD_p_, particularly in the IPWP, ITCZ and equatorial Indian Ocean (Figures 4e and 3f).

**Table 1:** Global Mean Surface Temperature (GMST)

Experiment	GMST (°C)	ΔSST (°C) W–E	Max ω anomaly (Pa/s)	ΔSST (°C) Trop. – Subtrop.	Ψ (kg/s)
Pre‐industrial	14.13	2.37	0.044	6.61	1.85e11
Abrupt 4 × CO_2_	20.15	1.31	0.038	6.13	1.65e11
Early Pliocene	18.99	0.47	0.025	3.99	1.35e11
Late Pliocene	17.85	2.17	0.041	6.23	1.76e11

*Note.* ΔSST in the tropical Pacific (8°S–8°N), West (130°E−155°W) minus East (155°W–80°W). Max tropical Pacific ω anomaly, calculated as the max value from 10°S to 10°N and 130°E−90°W with the mean value (from 10°S to 10°N) removed, meant to represent the strength of the Pacific Walker circulation. ΔSST between the tropics (15°N–15°S) and subtropics (20°N/S–40°N/S). Difference between maximum and minimum value of the atmospheric meridional overturning mass streamfunction (Ψ, kg/s), meant to represent the strength of the Hadley cell.

There are several tropical and subtropical regions that display consistent differences in δD_p_ when contrasting the EP SST patterns against the response to the A4X and LP SST patterns (Figures 4a and 4b). These are taken to represent the regions where the weaker zonal and meridional SST gradients in the EP simulation have the largest effect. We focus on examining two regions (black boxes, Figure 4) that have the biggest signals in these simulations, as they may be detectable within land‐based water isotope proxy records: (a) the equatorial Pacific and (b) the Sahel region in North Africa. In these regions, the differences simulated in δD_p_ are consistent across both pairs of experiments, despite the differences in global mean surface temperature between A4X and the LP (Table 1).

### 3.1 The Equatorial Pacific

Convective mass fluxes have been shown to decrease under global warming scenarios (Held & Soden, 2006). This reduction in convective mass flux should be reflected in the equatorial Pacific, as the region is characterized by strong moisture convergence and deep convection. Furthermore, enhanced extratropical warming reduces the supply of relatively cold water to equatorial upwelling regions in the eastern Pacific leading to a reduction in the zonal SST gradient and Walker Cell (Burls & Fedorov, 2014b; Heede et al., 2020). As shown in Table 1, there is a weakening of both the Walker and Hadley circulations in all three warming scenarios, with dramatic weakening in the EP due to the additional influence of a greatly reduced meridional SST difference between the tropics and the mid‐latitudes, and zonal SST differences between the eastern and western equatorial Pacific. A weakening Walker circulation should have opposite effects on the hydrological cycles—and therefore the isotopic signals—of the western and eastern Pacific. A reduction of rising (descending) air should result in a reduction (increase) of all aspects of the amount effect. In the IPWP, where convective uplift is weakened, there will be less low‐level convergence of depleted air, less convective precipitation, and less deep convective mixing. All these factors should enrich the isotopic ratio of both the lower tropospheric vapor and precipitation. Likewise, in the central and eastern Pacific, where the subsiding arm of the Walker circulation has been weakened, more and deeper convection occurs (Dee et al., 2018). The increase in convection depletes the δD_v_ and δD_p_. In this respect, changes in isotope ratios driven by the amount effect can be thought of as a dynamic effect, rather than thermodynamic.

**Figure 5:**
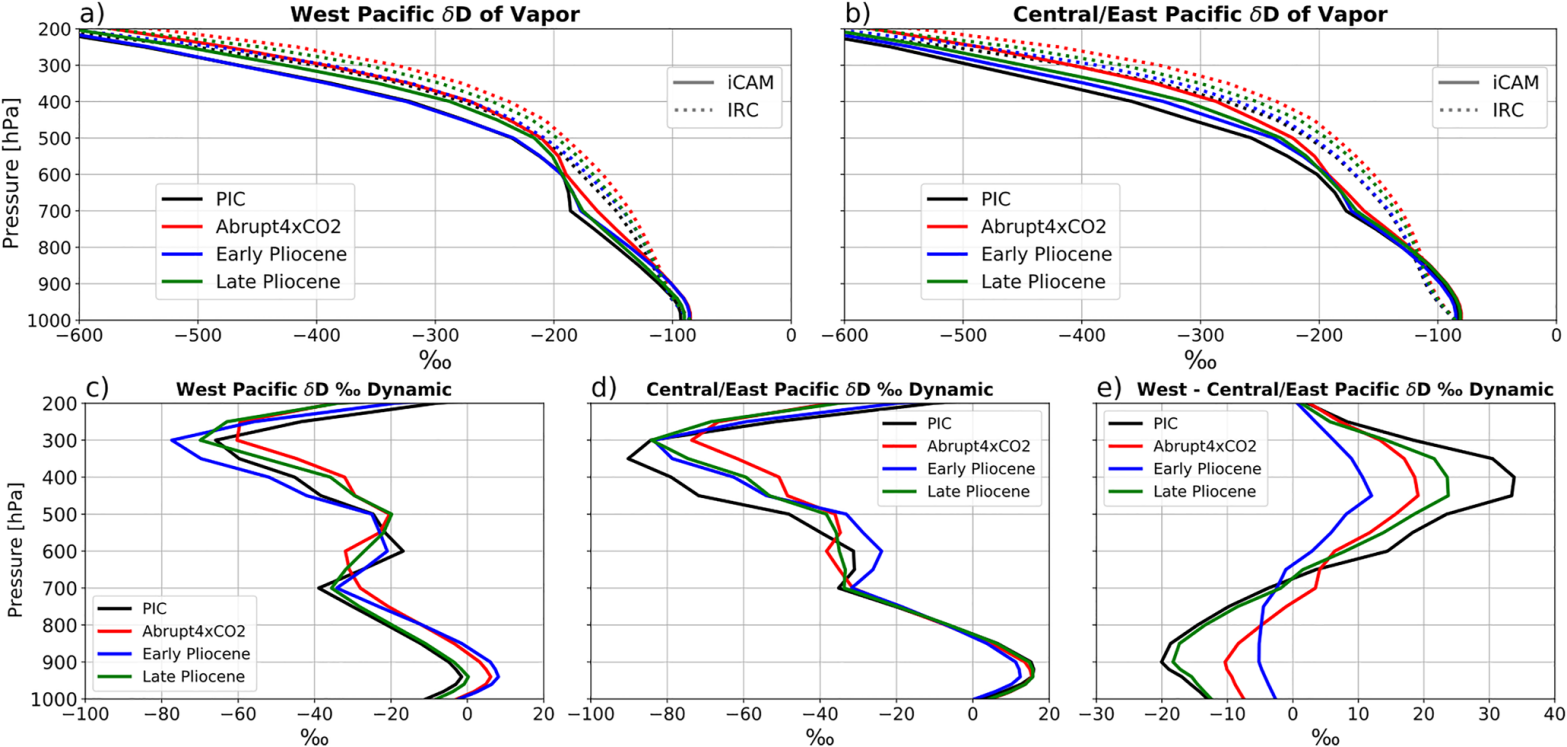
(a) The vertical profile of the 𝛿D of vapor in the Idealized Rayleigh Column (IRC; dotted lines) and the iCAM output (solid lines) in the west Pacific (15°S–15°N, 90°E−160°E). (b) As in (a), but for the central/east Pacific (15°S–15°N, 170°E−90°W). (c) The dynamic component profile in the west Pacific, calculated as the residual between the IRC and the iCAM output. (d) As in (c), for the central/east Pacific. (e) The difference between (d) and (c).

**Figure 6:**
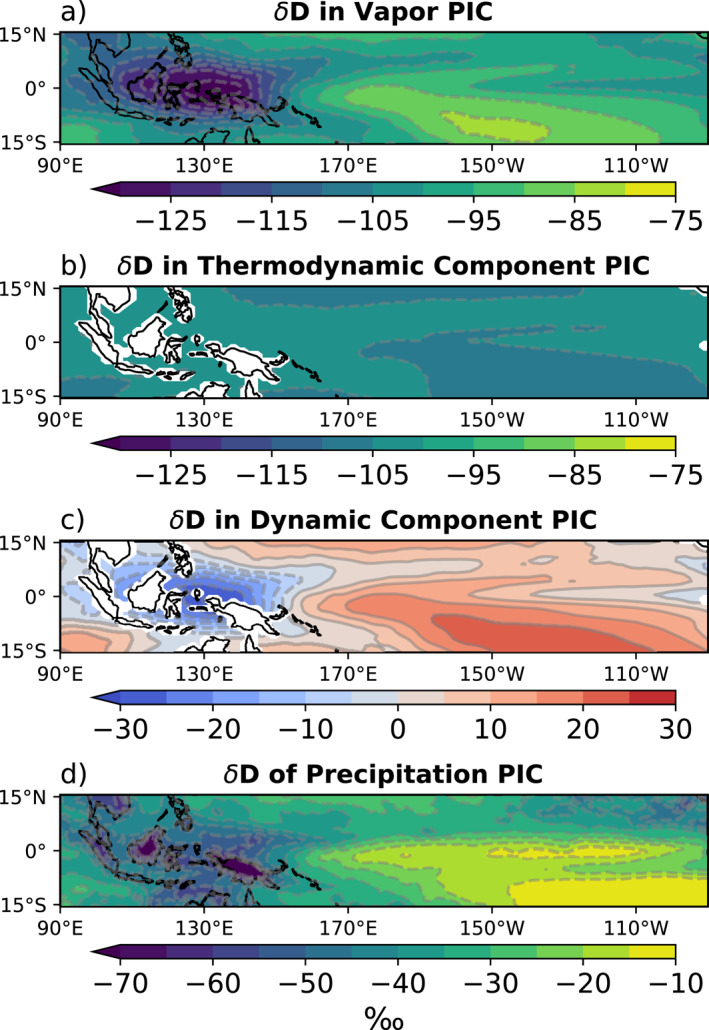
Lower Tropospheric (850 hPa—surface) pre‐industrial control 𝛿D values in (a) vapor, (b) thermodynamic component of idealized Rayleigh column model, (c) the difference between the two (a)–(b) that is, the dynamic component, and (d) precipitation. Land grid cells have been excluded from calculations of the thermodynamic and dynamic effects (b) and (c).

To examine the relative contributions of the thermodynamic and dynamic effects to the distribution of water isotopes, we adopt the idealized Rayleigh column (IRC) model (Dee et al., 2018). The IRC identifies the maximum theoretical depletion to the δD of the vapor column due to the temperature profile at each grid (the thermodynamic effect, Figures 5a and 5b). The residual between the actual vapor content output from the model and the calculated thermodynamic contribution is called the dynamic contribution, which represents changes to the δD_v_ of the column due to the effects of atmospheric circulation on condensation and evaporation (Figures 5c and 5d). The thermodynamic and dynamic effects controlling the 𝛿D field of lower tropospheric vapor (δD_v_) largely control the δD_p_ field, as evidenced by the similarity in both spatial patterns and magnitudes of the 𝛿D of lower tropospheric vapor and the δD_p_ (Figures 6a and 6d).

This can be seen within the IRC framework by isolating regional averages over the west and east equatorial Pacific, respectively (Figure 5). Figures 5c–5e shows that as the strength of the Walker circulation decreases from the PIC to the LP, the LP to the A4X, and the A4X to the EP (Table 1), the lower Tropospheric dynamic effect acts progressively to enrich water isotopes in the west and deplete water isotopes in the east (and vice versa for the upper Troposphere).

The IRC decomposition for the PIC at each grid point in the lower troposphere shows that the thermodynamic component has little spatial structure, but accounts for much of the magnitude of δD_v_ in the lower troposphere (Figures 6a and 6b). The dynamic component is much smaller in magnitude, but explains nearly all of the spatial structure seen in the vapor (Figure 6c).

**Figure 7:**
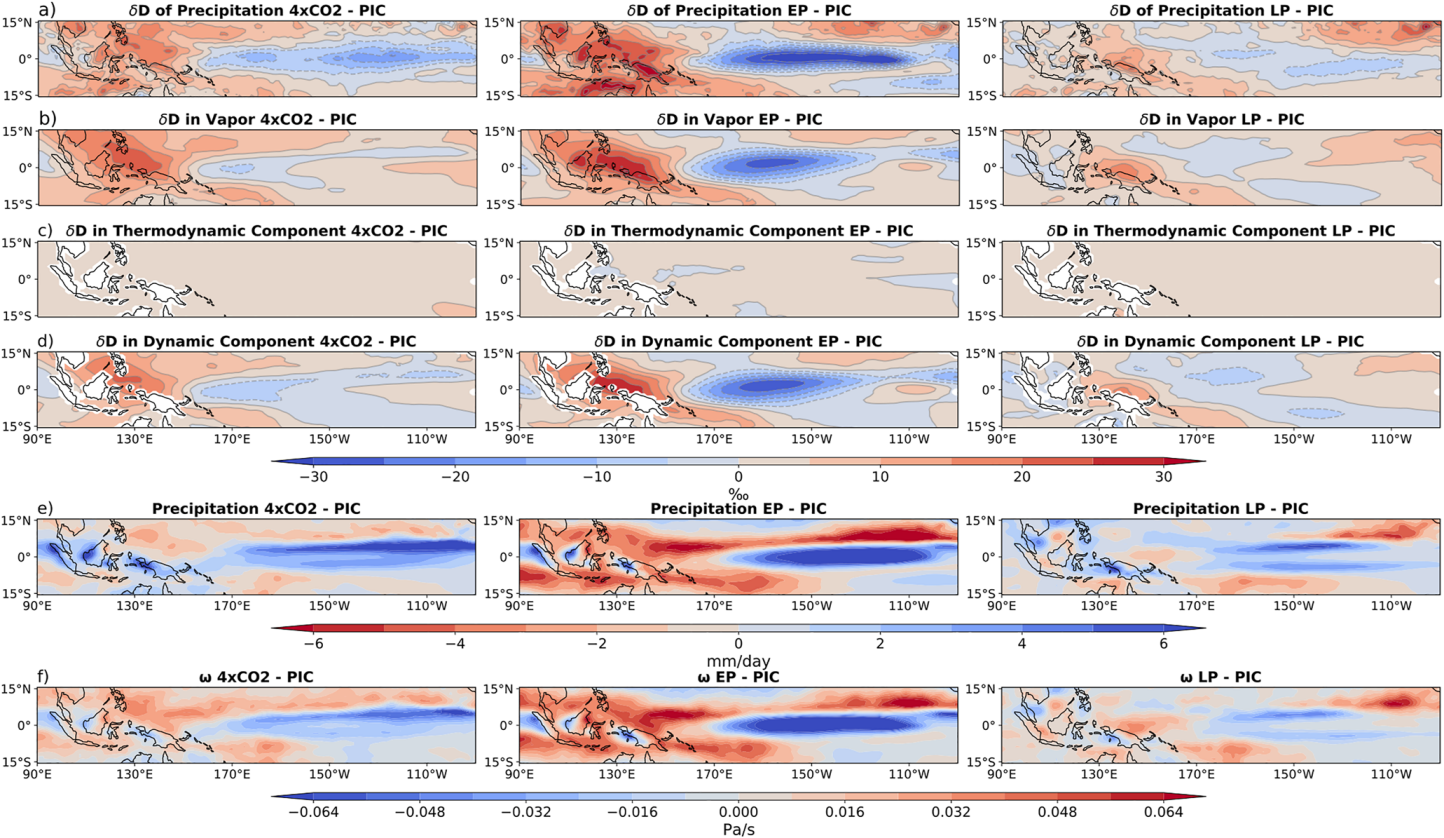
Warming scenario anomalies relative to PIC. (a) 𝛿D of precipitation. (b) 𝛿D of vapor averaged from 850 hPa to the surface. (c) How much of the 𝛿D of vapor is attributed to the thermodynamic component, according to the IRC model. (d) 𝛿D of vapor due to the dynamic component, which is the difference between (b) and (c). (e) Precipitation in mm/day (f) vertical velocity averaged over 250–500 hPa in Pa/s.

Applying this to the warming scenarios, it is possible to deduce how the IRC decompositions change in relation to the PIC (Figure 7). The results are similar to those in Figure 6, in that the thermodynamic terms are nearly spatially homogeneous, while the dynamic terms account for the majority of the spatial differences in the water isotope fields. However, the magnitude of the differences in the dynamic term are far larger than those of the thermodynamic term (Figure 7c vs. Figure 7d). The close match between the differences in dynamic terms and the differences in δD_p_ support the notion that differences in dynamics are largely responsible for the differences seen in tropical Pacific δD_p_ in the warming scenario simulations (Figures 7a and 7d).

In order to identify which processes are accounting for the dynamic changes in the warming scenarios, we plotted the changes in precipitation (Figure 7e) and the changes in vertical velocity (Figure 7f). Changes in precipitation amount and the strength of deep convection represent different aspects of the amount effect, which appear to explain the dynamic component. In all three scenarios, we observe a decrease in precipitation over the IPWP, along with an increase over the central and eastern Pacific. The upper Tropospheric vertical velocity has a nearly identical pattern, with decreases (increases) in vertical velocity coinciding with decreases (increases) in precipitation. These changes in deep convective precipitation are consistent with a weakening of the Walker circulation; indeed, the magnitudes of the changes between experiments correspond to the amount of Walker circulation weakening given in Table 1.

### 3.2 The Sahel

We next consider the West African Monsoon (WAM) and isotopic compositions in rainfall over the critically water‐stressed region of the Sahel. We cannot use the IRC model for this region, as that model assumes an oceanic lower boundary and a nearly moist adiabatic lapse rate, neither of which is applicable over the African continent. The wet season of the WAM occurs during Boreal summer, when the large temperature difference between the North African continent and the equatorial Atlantic to the south shifts heavy precipitation from the Gulf of Guinea northwards into the Sahel. To evaluate how well iCAM5.3 reproduces the WAM, we compare seasonal precipitation in the PIC to the Climate Hazards Group Precipitation Climatology (CHPclim) data set. The CHPclim data set is a high resolution (0.05°) monthly precipitation climatology product which combines infrared and microwave satellite data with a geospatial modeling approach based on moving window regressions and inverse distance weighting interpolation (Funk et al., 2015). For comparison, the CHPclim data are regridded to the lower resolution iCAM grid.

**Figure 8:**
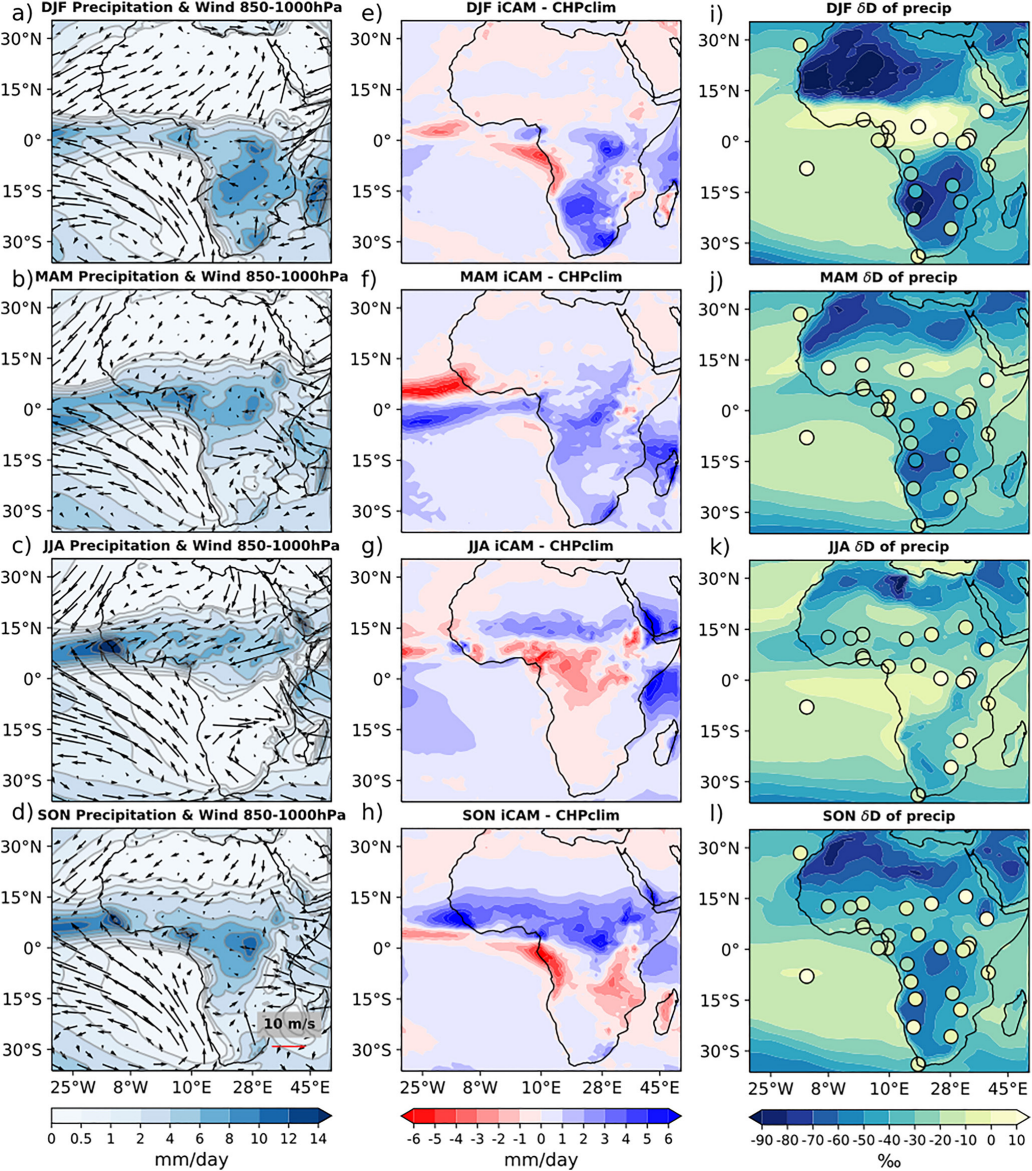
(a)–(d) Seasonal precipitation and average 850–1000 hPa wind in the PIC. (e)–(h) Seasonal precipitation in the PIC minus CHPclim. (i)–(l) Colored contours show seasonal 𝛿D_p_ in the PIC, colored circles show seasonal 𝛿D_p_ from GNIP station data.

**Figure 9:**
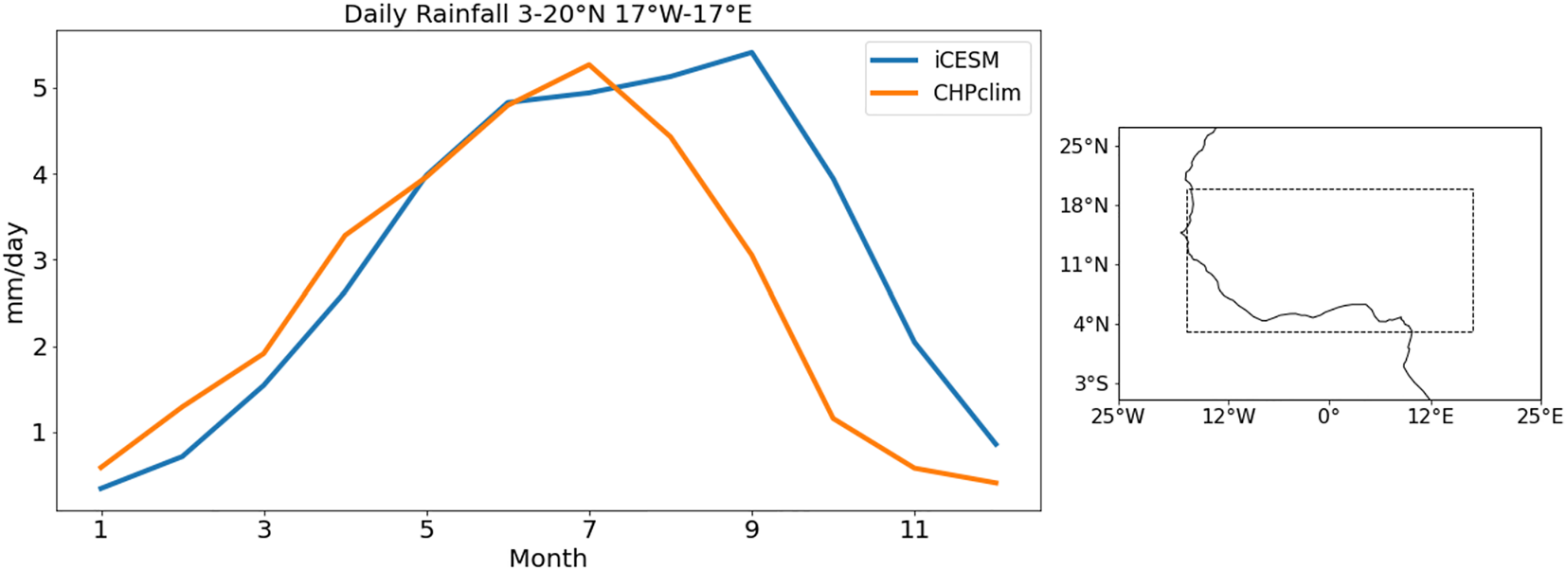
Average daily precipitation (left) in the boxed Sahel region (right) in iCAM5.3 (from the PIC simulation) and CHPclim.

A comparison of the mean seasonal precipitation over Africa in the PIC and CHPclim reveals seasonal biases present in iCAM (Figures 8e–8h). During the periods of most intense rainfall in JJA and SON, there is a clear northward bias in iCAM (Figures 8g and 8h). A large part of that bias is due to the maximum precipitation in iCAM remaining roughly 2 months longer than the July peak present in CHPclim (Figure 9).

To examine the impact of precipitation biases on δD_p_, we compare the δD_p_ in the PIC to station data from the Global Network of Isotopes in Precipitation (GNIP) IAEA/WMO (2021) (Figures 8i–8l). Seasonal station data is plotted from a station if there are at least 3 years of data for a given season, from the period 1953–2021. Overall, the model values are too negative at every station. However, we find no correlation between the precipitation bias in the PIC at the grid point nearest to each station and the δD_p_ bias in the same grid. This does not mean that precipitation biases are not important for δD_p_ biases. Isotopic values integrate the effects of atmospheric processes from source to sink, so bias at a single point is unlikely to be responsible for a majority of the signal. In DJF and MAM over central and southern Africa (Figures 8i and 8j), the model generally agrees with the GNIP spatial patterns of δD_p_.

**Figure 10:**
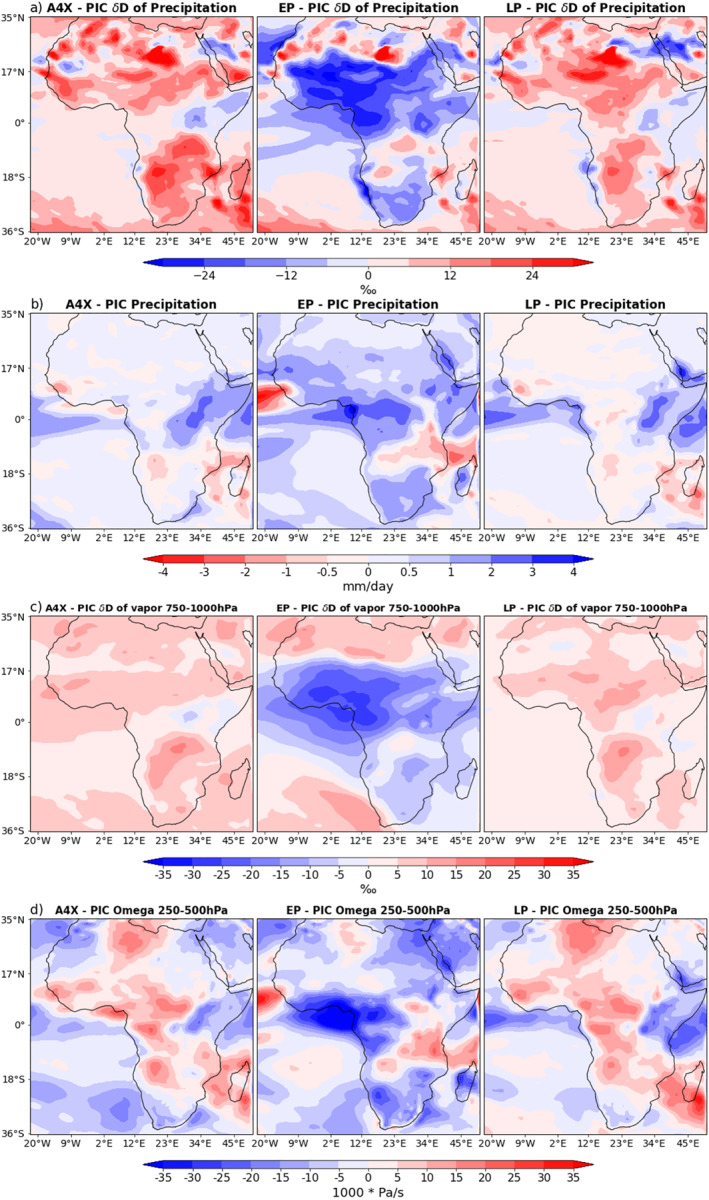
For each experiment, annual means of (a) 𝛿D of precipitation, (b) daily precipitation, (c) upper Tropospheric (250–500 hPa) vertical velocity, (d) 𝛿D of vapor and wind vectors averaged between 750 and 1,000 hPa.

The signal of reduced δD_p_ in the EP relative to the LP and A4X is consistent in the Sahel region (Figures 4a and 4b). The δD_p_ fields in Figure 10a highlight this feature, while also illustrating how similar the A4X and LP are despite their differences in SST forcing (Figures 1b and 1d). The anomalous precipitation patterns (Figure 10b) do not line up with the δD_p_ changes as cleanly as in the Pacific. Nevertheless, there is general correspondence between the precipitation changes (Figure 10b) and the δD_p_ changes (Figure 10a) across tropical Africa within each simulation. The EP is wetter over most of the African continent relative to the other two warming scenarios, which contributes to the relatively low δD_p_. Much of the area of reduced δD_p_ in the EP is also covered by regions of anomalous deep convection (Figure 10d), which further contributes to the depletion of vapor and precipitation. Additionally, the winds blowing north from the Gulf of Guinea in the PIC (Figures 8b–8d) are representative of the winds in the warming experiments (not shown). The vapor imported on shore by these winds has much lower isotopic values in the EP than the A4X and LP (Figure 10c).

### 3.3 Precipitation Proxy Comparison

We summarized all available Pliocene‐aged plant wax 𝛿D precipitation archives (*n* = 8 marine cores, *n* = 1 lake core), these data are only available for Africa and, with no data in the rest of the world other than two sites in the Bengal Fan (Table 2). We averaged available data for the two periods of interest: 5‐4 Ma (early Pliocene), and 3.3–3.1 Ma (late Pliocene). We note 𝛿D values are relatively invariant within Africa and close to the precipitation isotopic composition expected for landfalling storms or evaporatively D‐enriched precipitation, or that following continental recycling in low latitudes. There are no extreme D‐depletion signals associated with deep convection or orographic distillation. Values for the Bengal Fan are slightly more D‐depleted as would be expected in the wetter climate. High latitude and high elevation D‐depletion is entirely unsampled by the available proxy archives.

**Table 2:** Pliocene Plant Wax‐Derived Precipitation Isotope (δD_p_) Values, Listing all Available Records From African Margins (*n* = 8 Records), and the Rest of the World (*n* = 1) in Chronological Order of Publication

					δD_p_ (‰)					δD_p_ (‰)			
Site	Location	Tran‐sport		Span (Ma)	Mean	sem	n	Span (Ma)		Mean	SEM	*n*	References
	*African margins*												
ODP 722	Arabian Sea	Wind		4–5	−39	4	12	3.1–3.3		−40	8	12	Huang et al. (2007)
ODP 1085	SE Atlantic Ocean	Wind		4–5	−63	5	6	3.1–3.3		−62	8	6	Dupont et al. (2013)
DSDP 231	Gulf of Aden	Wind		4–5	−43	2	41	3.1–3.2		−35	5	41	Liddy et al. (2016)
DSDP 241	W Indian Ocean	Wind		4.79	−46	11	1	–		–	–	–	Polissar et al. (2019)
ODP 659	E Atlantic Ocean	Wind		4.03–4.88	−44	8	2	–		–	–	–	Polissar et al. (2019)
ODP 959	E Atlantic Ocean	Wind		4.13	−40	11	1	–		–	–	–	Polissar et al. (2019)
HSPDP‐BTB13	Lake Baringo, KE	Fluvial		–	–	–	–	3.1–3.2		−39	9	1	Lupien et al. (2020)
IODP U1478	SW Indian Ocean	Fluvial		4–4.05	−33	5	7	3.1–3.3		−33	4	7	Taylor et al. (2021)
	*Rest of the World*												
IODP U1445	Bay of Bengal	Fluvial		4–5	−71	6	5	3.1–3.12		−86	9	5	Dunlea et al. (2020)

*Note.* δD_p_ values are derived from the reported plant wax δD values and converted accounting for a net fractionation of −100‰. For each marine or lake core, the time span and number of data points are presented for each of the two periods of interest. Please note these are all the published δD plant wax reconstructions for the Pliocene, all are from around Africa and the Bay of Bengal. This proxy synthesis highlights the low resolution of many Pliocene records and a data void for much of the world.

We connect the proxy data to a source region based on a reasoned understanding of plant wax sourcing and transport pathways. Evidence from large river system indicates an exponential decline in distance‐sourcing, such that most plant wax is derived from the land within the nearest 500 km of the mouth of the river (Eglinton et al., 2021), and lowland sources dominate the export from large river networks like the Ganges‐Brahmaputra (Galy et al., 2011). Although catchment integration may vary with the detailed vegetation, rainfall and erosional/depositional settings with river networks, and that these may change with climate change over time (Usman et al., 2018), a local sourcing integrating a radius of 600 km from the coast is the approximation used in this study to allow for proximal fluvial sourcing within the river catchment and some coastal wind delivery as well. Source regions maybe much larger in wind‐dominant erosion regimes where dust plumes (Prospero et al., 2002) are shown to entrain plant wax over long‐distance export readily transporting over thousands of km (Rommerskirchen et al., 2003; Schefuss et al., 2003, 2009). We factored in geological setting, especially fluvial or wind‐transport, major dust source regions (such as the Bodele depression and Afar Triangle) and dominant wind directions to provide a custom assessment of source regions. These custom source regions were used to estimate modeled 𝛿D of precipitation contributed to each site, taking the areal average of the model output in each of the defined source regions.

**Figure 11:**
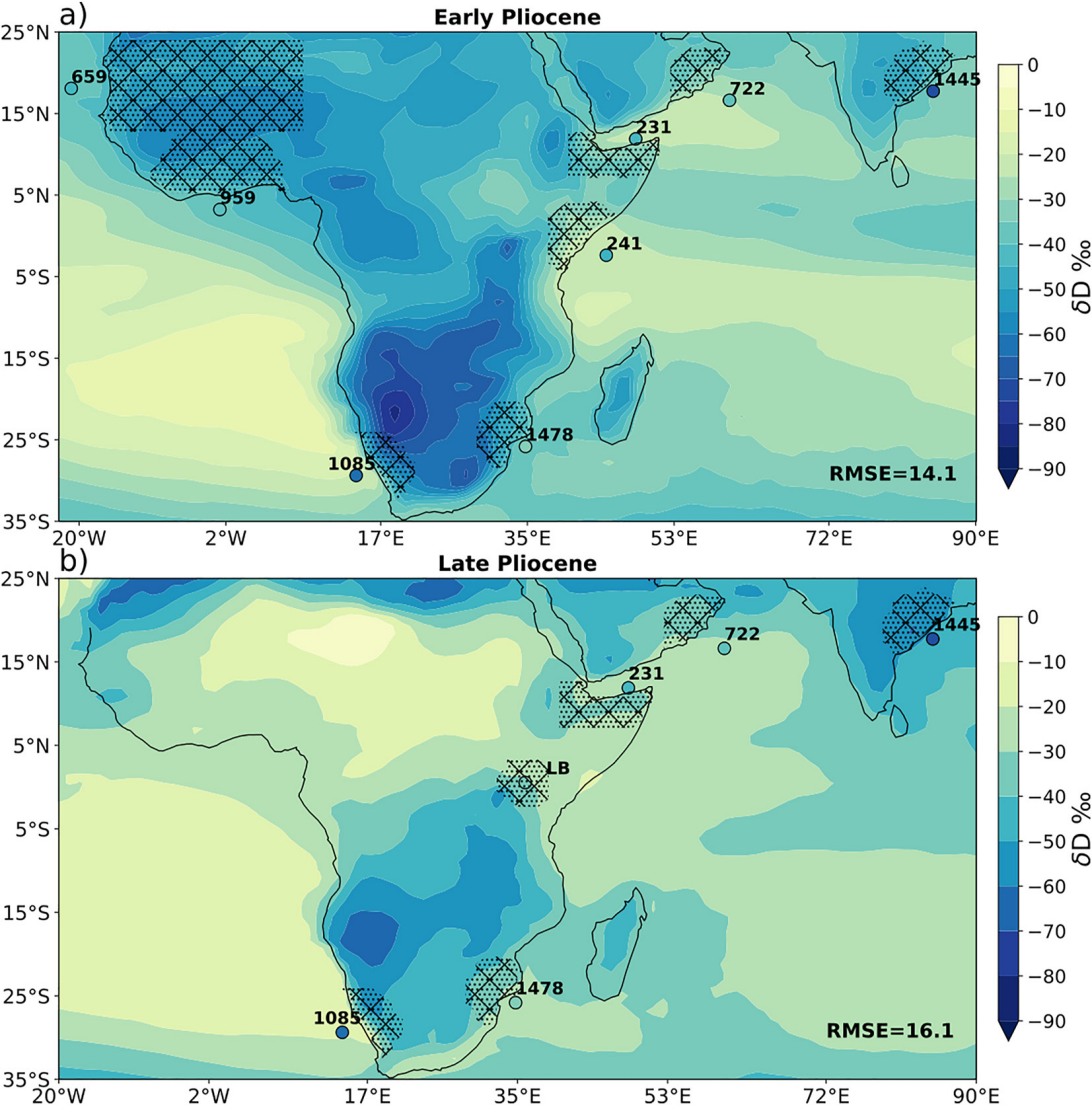
Model‐simulated (a) early Pliocene and (b) late Pliocene precipitation 𝛿D (map shading) compared to plant‐wax proxy estimates of 𝛿D_p_ (colored circles; labeled with abbreviated DSDP/ODP/IODP marine core site number, LB = Lake Baringo). Hatched areas represent defined source regions for each site. RMSE is calculated by comparing the average modeled precipitation isotope value in the hatched region to the reconstructed proxy value for that site. Please note this map view is an incomplete view of the world, but encompasses all published Pliocene δDp archives.

Plant wax 𝛿D based precipitation 𝛿D averages for each site and time slice (Table 2, Figure 11, colored circles) were compared to the model estimated precipitation 𝛿D from the inferred plant wax source regions (Figure 11, hatched regions) for the EP and LP scenarios (Figure 11). In addition, we compared proxy data to the pre‐industrial control and abrupt 4 × CO_2_ iCAM simulations (not shown). As most Pliocene archives are marine core we capture a dominantly coastal signature, leaving modeled D‐depletion inland unsampled and untested by proxy data, although this continental effect is minimal in Africa, the problem would be more extreme in other parts of the world including high elevation continental interiors.

We calculated the average proxy‐model δD_p_ divergence relative to all four model scenarios, determining the root mean squared error (RMSE) between all of the modeled source regions and the respective proxy derived value is computed for each model and both time periods (Table 3). We find that the EP scenario has the lowest RMSE with the Early Pliocene proxy data, and the LP scenario has the lowest RMSE with the Late Pliocene proxy data, suggesting that the intended simulations appear to represent the precipitation isotope data well. However, a Student’s *t*‐test between each pair of squared errors shows that none of the models have errors which are significantly different from one another at the 5% significance level. The lack of significant difference between proxy‐model comparison for the various model scenarios, leaves the search for the best fit Pliocene scenario as inconclusive at this time. Thus we cannot differentiate between the SST gradients, and their precipitation isotopic “fingerprints” in the model, based on the available proxy δD_p_ data. We need more proxy to achieve robust differentiation of which model scenario best fits the proxy data, and we encourage prioritization of the regions (maritime continent and Sahel) that show strong responses to the choice of SST gradient in our simulations (the so‐called precipitation isotope “fingerprints” of SST changes).

**Table 3:** Proxy‐Model Comparison of Proxy and Modeled δD_p_ Values, Tabulating the Divergence Between Proxy and Model as Root Mean Squared Error (RMSE) Between all Available Precipitation Isotope Proxies for Each Time Slice to Each Model Scenario

	Proxy‐model RMSE (‰) for each scenario			
Time slice	PIC	A4X	EP	LP
Early Pliocene (4–5 Ma)	16.2	17.6	**14.1**	15.6
Late Pliocene (3.2 Ma)	19.1	19.3	18.7	**16.1**

*Note.* Bold values highlight the best proxy‐model match in each time slice, the EP scenario best matches the early Pliocene proxy data and LP scenario best matches the Late Pliocene proxy data.

## 4 Discussion

### 4.1 SST Scenarios for Pliocene Simulations

By forcing iCAM5.3 with SST and sea‐ice fields thought to be representative of the early and late Pliocene, we have identified 𝛿D of precipitation signals in two regions as being dynamical fingerprints of weaker than modern large‐scale surface temperature gradients. Based on our findings, these two regions—the maritime continent and the Sahel—make strong candidates for targeted precipitation proxy collection and reconstructions. Our preliminary model‐proxy comparison makes use of plant wax precipitation isotope reconstructions from Pliocene sediment core archives (*n* = 9 sites) and hints at agreement in both the early and late Pliocene with our modeling results, despite the apparent negative bias in iCAM. However, more data are needed to claim a robust agreement. Such an agreement—or lack thereof—has the potential to provide an important piece of evidence in the effort to assess the degree to which the atmospheric dynamics resulting from Pliocene SST gradient reconstructions can explain hydrological cycle reconstructions.

### 4.2 Fingerprint Region 1: The Maritime Continent

The equatorial Pacific is expected to show changes related to the weakening Hadley and Walker circulations in response to the reduced zonal SST difference in the late Pliocene and greatly reduced zonal SST difference in the early Pliocene SST field. The weakening Walker circulation is shown to be the dominant control on the water isotope ratio distribution in the region, which is characterized in the EP by higher isotopic values in the IPWP and lower isotopic values in the central and eastern equatorial Pacific. Applying the IRC model shows that the thermodynamic effects are spatially homogeneous, while dynamic effects largely control the spatial isotopic distribution. The dynamic effect is shown to be due to changes in precipitation and convection, or the amount effect, in response to a weakening of the Walker Circulation. Should precipitation proxy data be collected from the maritime continent (many island landmasses with glacially exposed shallow continental shelves) within the IPWP region, we expect to see a gradual trend toward more negative δD_p_ through time from the EP, as the SST gradients and Walker circulation strengthen.

### 4.3 Fingerprint Region 1: The Sahel

Due to the limitations of the IRC model, the relative contribution of dynamic and thermodynamic terms to changes in δD_p_ over the Sahel cannot be as easily diagnosed. The large reduction in δD_p_ over the Sahel in the EP appears to be related to differences in precipitation and deep convection. The early Pliocene simulations yield wetter conditions (more precipitation) over the Sahel, associated with increased deep convection, and attendant D‐depletion. Moisture sources over the Gulf of Guinea also experience more D‐depletion from increased deep convective precipitation, so that the moisture advected toward the continent has lower δD_v_ in the EP.

Although the only available Pliocene precipitation proxy records are located in and around Africa, only two sites are near the Sahel. More data are needed to evaluate the modeled fingerprint.

### 4.4 Model Limitations and Recommendations for Future Experimentation

While not specifically addressed here, there appear to be other regions affected by SST gradient changes that deserve further attention, such as the Andes region of South America, however this region is discounted from consideration here because of the complication of adequate representation of topography within climate model data that would merit a distinct approach. A full exploration of all possible sites is beyond the scope of this paper, and could be the subject of future regionally focused model‐proxy comparisons.

We acknowledge important limitations of this work. Modern land‐surface boundary conditions are used for all experiments, to isolate the effects of SST forcing alone on the simulated changes in isotopic distributions. A more realistic simulation of Pliocene conditions would require knowledge of Pliocene boundary conditions, such as vegetation and orography. Changing vegetation types and distributions could have affected albedo, surface temperature, sensible and latent heat fluxes, continental recycling of precipitation (with attendant isotope effects), and local aerosol loading, each of which may affect climate in ways untested here. In the case of the mid‐Piacenzian, wetter conditions in the Sahel and east Asia are largely driven by vegetation and ice sheet changes (Feng et al., 2022). Each of these topics would require extensive work to constrain using proxy data and model experimentation. However there are clues from other time periods that these boundary conditions may be significant. In simulations of the Holocene “Green Sahara” event, many models fail to generate monsoon precipitation in the Sahara matching proxy reconstructions, likely due to incorrect vegetation feedbacks and too much dust (Pausata et al., 2016; Tierney et al., 2017). The exact effects of Pliocene paleovegetation on δD_p_ are unknown and would ideally be considered in further efforts to model Pliocene climate and conduct a model‐proxy comparison using isotope‐enabled climate models.

### 4.5 Proxy Limitations and Recommendations for Future Reconstructions

Although the number of proxy records of precipitation isotopes for the Pliocene has been growing (Table 3), we lack sufficient data points to robustly constrain maps of precipitation isotopes for time slices of interest for proxy‐model comparisons. In addition to data gaps for much of the world outside of Africa and the Bengal Fan for the Pliocene, there are also questions of data density within the available sites. Some sites have very few datapoints to constrain temporal variability and some lack sufficient age control to make detailed assessments of glacial‐interglacial or precession phasing that may matter for precipitation isotopes. As demonstrated in Prescott et al. (2014), Pliocene surface air temperatures around Africa are sensitive to orbital parameters, varying by several °C. This raises the question as to whether the orbital signal of δD_p_ is larger than the signal due to the slowly evolving mean SST gradients. While most age models are insufficient to demonstrate orbital pacing, Liddy et al. (2016) and Taylor et al. (2021) have timeseries that approximately resolve precessional pacing, showing that the “likely precessional” variability is around 10–20 per mil.

We present all data as originally published, and additional effort could be warranted with sufficient investment in additional data collection to enable more detailed proxy‐model comparison efforts. What we present here is the state of present knowledge and this is we believe the first such effort focused on precipitation isotopes for any time slice.

Using the simulations here, motivated by the question of Pliocene SST gradients, we identify two priority regions for additional data—the Sahel and the IPWP, or rather the maritime continent land mass where plants grow. In addition isotopic fingerprints were detected in the Andean region of South America, although topography presents additional complications there. Other pressing priorities for regional hydroclimate reconstructions can be identified. For example, to test orographic uplift questions for the Pliocene or to test regional climate change issues in water scarce regions, such as the question of water availability in southwestern North America. We recommend integration of proxy‐model thinking into archive collection and reconstructions using the plant wax δD proxy in future.

## 5 Conclusion

We have identified two regions (the Sahel and the IPWP) where the simulated δD of precipitation change is distinct in an early Pliocene SST reconstruction, relative to a late Pliocene reconstruction and a 4 × CO_2_ scenario. The differences in these two regions are due to changes in both circulation and precipitation amount, rather than purely thermodynamics. A model‐proxy comparison with nine sites in and around Africa indicates that the early and late Pliocene modeled δD_p_ fields are the best match to their respective proxy data, but more data are needed for a robust test of the Sahel fingerprint in particular. Additional plant wax δD proxy reconstructions spanning the Pliocene, a key analog for future climate change, are sorely needed to evaluate the dynamical consistency between Pliocene SST and hydrological cycle reconstructions globally. We emphasize the relevance of Sahel and maritime continent reconstructions to help constrain fingerprints of Pliocene reduced zonal and meridional SST gradient changes. In addition, higher latitudes would be high priorities to test the implications of meridional SST gradient changes and the degree of high latitude warmth via the temperature effect. Additional regional priorities may guide other proxy reconstructions, include water stressed highly populous regions like the southwestern North America, and much of the world beyond Africa and South Asia has no proxy reconstructions of precipitation isotopes using the plant wax δD proxy. If precipitation isotope reconstructions can help constrain hydrological cycle representation in climate models that would help to build confidence in future rainfall projections in climate models. Generating higher confidence in Pliocene SST reconstructions will facilitate better use of Pliocene “scenario” climate model simulations for insight into modern climate change. This work will not only address the SST debate for the Pliocene but also tests the dynamical basis for regional hydrological cycle changes in the past and thus build confidence in model projections for the future.

## Supporting information

Supporting Information S1

## Data Availability

The Jupyter Notebook to execute the analysis in the paper is preserved with Zenodo (Knapp, 2022). The SST forcing files, model output used for analysis, GNIP data, CHPclim data, and plant wax data are archived with Zenodo (Knapp et al., 2022).
