# Supplementary material for: A Pliocene Precipitation Isotope Proxy‐Model Comparison Assessing the Hydrological Fingerprints of Sea Surface Temperature Gradients
Source: Paleoceanogr Paleoclimatol. 2022 Dec 24;37(12):e2021PA004401. doi: 10.1029/2021PA004401 (PMC10108060; doi:10.1029/2021PA004401)
Supplement: Supplementary file 1 — Supporting Information S1 [file PALO-37-0-s001.docx]

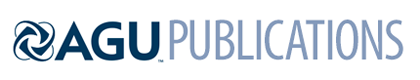


*Paleoceanography and Paleoclimatology*

Supporting Information for

**A Pliocene precipitation isotope proxy-model comparison assessing the hydrological fingerprints of sea surface temperature gradients**

Scott Knapp^1*^, Natalie J. Burls^1^, Sylvia Dee^2^, Ran Feng^3^, Sarah J. Feakins^4^, Tripti Bhattacharya^5^

^1^Department of Atmospheric, Oceanic, and Earth Sciences, George Mason University, Fairfax, VA 22030, USA.

^2^Department of Earth, Environmental and Planetary Sciences, Rice University, Houston, TX 77005, USA.

^3^Department of Geosciences, University of Connecticut, Storrs, CN 06269, USA.

^4^Department of Earth Sciences, University of Southern California, Los Angeles, CA 90089, USA.

^5^Department of Earth and Environmental Sciences, Syracuse University, Syracuse, NY 13902, USA.

*Corresponding author: Scott Knapp ([sknapp4@gmu.edu](mailto:sknapp4@gmu.edu))

**Contents of this file**

Text S1

Table S1

Figure S1

**Introduction**

- Text S1 details the interpretation of precipitation isotopic proxies from plant wax
- Table S1 has the absolute dD values of the modelled source regions to compare to the proxy derived dD values
- Figure S1 shows the modelled wind patterns around Africa

Text S1.

Interpretations of the precipitation isotopic proxy information contained in plant wax δD values needs to account for the net or “apparent” fractionation, the balance of evapotranspiration and biosynthetic fractionation in plants. At the plant scale, a complex set of factors from plant ecohydrology, leaf physiology (e.g., grass versus tree) and biochemistry (e.g., C3 versus C4) may lead to a spread between plant leaves. However, the plant wax δD in sedimentary archives averages that of the plant community across the source region. Despite the large range in δD values reported in plant studies, the central estimates of large plant surveys and the natural integration provided by sediment tops reveal that on average fractionations are on the order of -93‰ in drylands (Feakins and Sessions, 2010; Feakins et al., 2019; Tierney et al., 2010) and -121‰ in tropical forests (Feakins et al., 2016) and in the temperate zones (McFarlin et al., 2019; Sachse et al., 2012). We considered the fractionations reported in the original Pliocene references, which often included some accounting for plant type (Dunlea et al., 2020; Liddy et al., 2016; Polissar et al., 2019). However other studies did not report a fractionation estimate (Dupont et al., 2013; Huang et al., 2007; Taylor et al., 2021), and the various data availability precludes a consistent vegetation-type correction. We took on a pragmatic approach and used a universal fractionation of -100‰, and a round number close to the value reported for African lake core tops (Tierney et al., 2010). This approach may overestimate the δD of precipitation by +7‰ in drylands and may underestimate the δD of precipitation by -21‰ in tropical forests.

**Table S1.**

| SITE | Span [Ma] | Mean dD_p_ | Control | A4X | EP | LP | Span [Ma] | Mean dD_p_ | Control | A4X | EP | LP |
| --- | --- | --- | --- | --- | --- | --- | --- | --- | --- | --- | --- | --- |
| African margins | | | | | | | | | | | | |
| ODP 722 | 4-5 | -39 | -29 | -40 | -35 | -36 | 3.1-3.3 | -40 | -29 | -40 | -35 | -36 |
| ODP 1085 | 4-5 | -63 | -41 | -34 | -55 | -40 | 3.1-3.3 | -62 | -41 | -34 | -55 | -40 |
| DSDP 231 | 4-5 | -43 | -27 | -30 | -36 | -30 | 3.1-3.2 | -35 | -27 | -30 | -36 | -30 |
| DSDP 241 | 4.793 | -46 | -22 | -24 | -30 | -23 | - | - | - | - | - | - |
| ODP 659 | 4.03 & 4.88 | -44 | -39 | -33 | -53 | -31 | - | - | - | - | - | - |
| ODP 959 | 4.1347 | -40 | -29 | -21 | -51 | -23 | - | - | - | - | - | - |
| IODP U1445 | 4-5 | -71 | -49 | -49 | -45 | -55 | 3.1-3.12 | -86 | -49 | -49 | -45 | -55 |
| HSPDPBTB13 | - | - | - | - | - | - | 3.1-3.2 | -39 | -27 | -33 | -42 | -31 |
| IODP U1478 | 4-4.05 | -33 | -40 | -32 | -51 | -36 | 3.1-3.3 | -33 | -40 | -32 | -51 | -36 |

**Table 1. Absolute values of proxy derived mean** δ**D_p_ at each site and the respective modelled source regions in the four experiments.**

**Figure S1.**


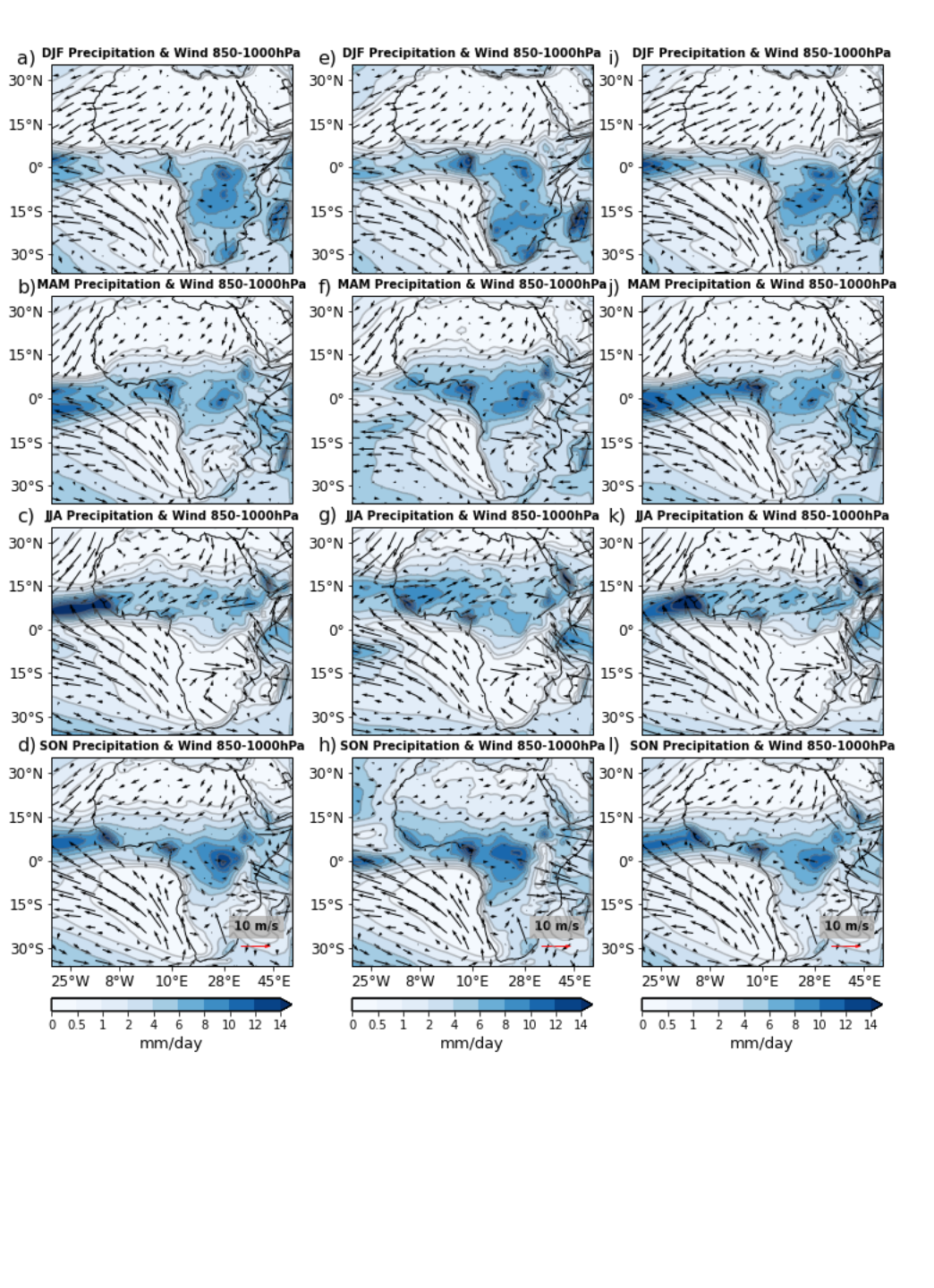


**Figure S1. Seasonal precipitation and average 850-1000 hPa wind in A4X (a-d), EP (e-h), and LP(i-l).**
